# Dietetic Treatment of Disease

**Published:** 1891-12

**Authors:** 


### Dietetic Treatment of Disease.

The physician often encouuters cases where little, if any, medicine is needed—but relies chiefly on nourishing the patient and conserving the vital forces. A knowledge then of the kind of food needed is of the highest importance. There are many kinds of prepared food in the market, and some are suited to one condition and some to another; doubtless they all have their proper sphere. Reed & Carnrick, the manufacturing chemists of New York, are known to be pioneer investigators in this field; and they have rendered the physicians valuable aid in having prepared several articles for food-stuffs. Amongst them Pancrobilin compound and tablet compound preparations are well known. This firm has published a book—an "*Abstract of Symptoms*," which they will send free, if this article be mentioned, to any physician. In this book not only are the different kinds of food described, but their physiological action is given,—the symptoms which indicate their use;—the diseases in which they are found most beneficial, ect. It contains a great deal of really valuable information, and is surely worth a letter and a two cent stamp; it is worth perusal and preservation for future reference.

